# Inhibitory effects of estrogen receptor beta on specific hormone-responsive gene expression and association with disease outcome in primary breast cancer

**DOI:** 10.1186/bcr1667

**Published:** 2007-04-10

**Authors:** Chin-Yo Lin, Anders Ström, Say Li Kong, Silke Kietz, Jane S Thomsen, Jason BS Tee, Vinsensius B Vega, Lance D Miller, Johanna Smeds, Jonas Bergh, Jan-Åke Gustafsson, Edison T Liu

**Affiliations:** 1 Genome Institute of Singapore, 60 Biopolis Street, #02-01, Singapore 138672, Republic of Singapore; 2 Department of Microbiology and Molecular Biology, Brigham Young University, 753 WIDB, Provo, UT 84602, USA; 3 Center for Biotechnology, Karolinska Institute, Hälsovägen 7-9, 141 57 Huddinge, Novum, Sweden; 4 Radiumhemmet, Karolinska Institute and University Hospital, S-171 76 Stockholm, Sweden; 5 Department of Biosciences and Nutrition, Karolinska Institute, Hälsovägen 7-9, 141 57 Huddinge, Novum, Sweden

## Abstract

**Introduction:**

The impact of interactions between the two estrogen receptor (ER) subtypes, ERα and ERβ, on gene expression in breast cancer biology is not clear. The goal of this study was to examine transcriptomic alterations in cancer cells co-expressing both receptors and the association of gene expression signatures with disease outcome.

**Methods:**

Transcriptional effects of ERβ overexpression were determined in a stably transfected cell line derived from ERα-positive T-47D cells. Microarray analysis was carried out to identify differential gene expression in the cell line, and expression of key genes was validated by quantitative polymerase chain reaction. Microarray and clinical data from patient samples were then assessed to determine the *in vivo *relevance of the expression profiles observed in the cell line.

**Results:**

A subset of 14 DNA replication and cell cycle-related genes was found to be specifically downregulated by ERβ. Expression profiles of four genes, *CDC2*, *CDC6*, *CKS2*, and *DNA2L*, were significantly inversely correlated with ERβ transcript levels in patient samples, consistent with *in vitro *observations. Kaplan-Meier analysis revealed better disease outcome for the patient group with an expression signature linked to higher ERβ expression as compared to the lower ERβ-expressing group for both disease-free survival (*p *= 0.00165) and disease-specific survival (*p *= 0.0268). These findings were further validated in an independent cohort.

**Conclusion:**

Our findings revealed a transcriptionally regulated mechanism for the previously described growth inhibitory effects of ERβ in ERα-positive breast tumor cells and provide evidence for a functional and beneficial impact of ERβ in primary breast tumors.

## Introduction

Estrogens are involved in a number of vertebrate developmental and physiological processes and have been implicated in certain types of endocrine-related tumors [1-4]. Hormone response in target tissues is mediated by nuclear receptors that function as ligand-dependent transcription factors. Receptor function is further modulated by post-translational modifications and interactions with other nuclear proteins. Originally, only one type of estrogen receptor (ER) was thought to be involved in hormone signaling. However, a second ER, termed ERβ, was subsequently discovered, adding another dimension of complexity to the regulation of hormone response. The original receptor was renamed ERα [5].

ERα and ERβ show 55% identity in their ligand-binding domains and approximately 97% similarity in the DNA-binding domains (DBDs). Both ERs bind estradiol with high affinity but vary in their ability to bind other natural and synthetic ligands and the types of response elicited upon ligand binding [6-8]. Reflecting the high degree of similarity in their DBDs, both receptors interact with the same conserved estrogen response element (ERE) (5'-GGTCAnnnTGACC-3') as either homodimers or α/β heterodimers [9-11]. Tissue-specific expression and co-expression of receptor subtypes suggest that ER homodimers and heterodimers may mediate distinct hormone responses [12-15]. Moreover, the discovery of ERβ variants with different structural and functional characteristics and tissue distribution further highlighted the potential complexity of the interactions between ERs and the mechanisms by which estrogen response is modulated [16-20].

The predominant impact of ER activation appears to be alterations in the transcriptional activity and expression profiles of target genes. A number of genes, including trefoil factor 1/pS2, cathepsin D, cyclin D1, c-Myc, and the progesterone receptor, are positively regulated by estrogen treatment [21]. Transcriptional repression by ER has not been as well studied. However, by means of SAGE (Serial Analysis of Gene Expression) and DNA microarrays, many more estrogen-responsive genes, induced or repressed by the hormone, have been identified and characterized [22-29]. Much of the work on gene expression has been focused on the role of ERα, but little is known about genes specifically targeted by ERβ or by α/β heterodimers. Recent microarray experiments using knockout animals indicate that target tissues in ERβ knockouts exhibited an overall increased transcriptional response to hormone treatment as compared to wild-type controls [30]. Expression studies of osteosarcoma cells stably transfected with each receptor subtype suggest that ERα and ERβ share some common target genes, although each receptor also appears to have distinct sets of downstream targets [31]. Despite these efforts, the exact transcriptional effects of ERα and ERβ in breast cancer remain obscure.

To characterize the impact of ERβ expression on hormone response in ERα-positive breast tumor cells, we have stably transfected T-47D (ERα^+^/ERβ^-^) cells with an inducible ERβ expression construct to generate subline T-47Dbeta. Induction of ERβ expression in this cell line was shown to inhibit estrogen-responsive cell proliferation [32]. These observations are consistent with other reports that describe the growth-inhibitory effects of ERβ [33,34]. Using high-density DNA microarrays under conditions that induce ERβ expression and following hormone treatment, we screened for potential transcriptional effects of the ERαβ co-expression. Here, we present a set of cell cycle and DNA replication genes responsive to ERβ expression and estrogen treatment and their potential roles in the biology of primary breast tumors.

## Materials and methods

### Cell culture

T-47D cells were cultured in a mixture of Dulbecco's modified Eagle's medium (DMEM) and Ham's F-12 1:1 supplemented with 5% fetal bovine serum (FBS), penicillin, and streptomycin. For experiments using 17β-estradiol, DMEM without phenol red and FBS treated with dextran-coated charcoal were used.

### Transfection and plasmids

T-47D cells stably transfected with tetracycline (TET)-regulated ERβ expression plasmid were generated in two steps: (a) The cells were first transfected with pTet-tTAk (Invitrogen Corporation, Carlsbad, CA, USA) modified to contain puromycin resistance using lipofectin according to manufacturer's instructions (Invitrogen Corporation). Selection was performed with 0.5 μg/ml of puromycin in the presence of 1 μg/ml of TET. A good clone showing a high level of induction and low basal activity was selected using the pUHC13-3 control plasmid (Invitrogen Corporation). The short form of ERβ encoding 485 amino acids was fused to the flag tag (ERβ485) and cloned into PBI-EGFP (Clontech, Mountain View, CA, USA). (b) This construct was then transfected into the previously described inducible clone together with a neomycin-resistance plasmid. The selection was performed using 500 μg/ml of G418 (Calbiochem, brand of EMD Biosciences, Inc., San Diego, CA, USA).

### Preparation of RNA

Cells were added to 150-mm plates at a confluency of 40%; after 1 day, the normal medium was replaced by the medium described above, supplemented with 5% dextran-coated charcoal-treated FBS (DCCFBS). After 24 hours, 10 nM ICI 182780 (fulvestrant) was added to the cultures and incubation proceeded for an additional 48 hours. For expression of ERβ, TET was removed 12 hours before initiation of treatment with 17β-estradiol. At time 0 hours, the medium was changed to 0.5% DCCFBS and 10 nM of 17β-estradiol or the equivalent volume of dimethyl sulfoxide for mock-treated controls was added. RNA was prepared by adding 10 ml of Trizol (Invitrogen Corporation) to each 150-mm plate at different time points after the start of treatment, and RNA was prepared according to the manufacturer's instructions.

### Microarray analysis

Microarrays were generated by spotting the Compugen USA, Inc. (Jamesburg, NJ, USA) 19 K human oligo library, made by Sigma-Genosys (The Woodlands, TX, USA), on poly-L-lysine-coated glass slides. Twenty-five micrograms each of sample total RNA and human universal reference RNA (Stratagene, La Jolla, CA, USA) were labeled with cyanine (Cy) 5-conjugated dUTP and Cy3-conjugated dUTP (PerkinElmer Life and Analytical Sciences, Inc., Waltham, MA, USA), respectively, and hybridized to the arrays using protocols established by the Patrick O. Brown Laboratory (Stanford University, Palo Alto, CA, USA) accessible at [35]. Array images and data were obtained and processed using the GenePix4000B scanner and GenePix Pro software (Axon Instruments, Inc., now part of Molecular Devices Corporation, Sunnyvale, CA, USA). A single sample from each time point and treatment condition was subjected to microarray analysis. Differentially expressed genes were determined using a 1.15-fold difference cutoff at multiple time points and were clustered and visualized using the Eisen Cluster and TreeView programs [36]. Gene ontology of responsive genes was derived from annotations (2002 version) made by Compugen USA, Inc. Significant enrichment of functional categories was determined by chi statistics by comparing the distribution of genes in a given functional category for all of the genes represented on the microarray (expected distribution) with the distribution of genes in the same functional category in differentially expressed genes (observed distribution). The *p *value was then calculated based on a two-tailed chi distribution, and a *p *value of less than 0.05 was considered a significant difference between the observed and expected values. The data are presented as the log2-transformed ratio between the treated/induced samples and the mock treated-uninduced controls for the same time point. The cell line microarray data have been deposited with the Gene Expression Omnibus under accession number GSE7206, and the normalized log2-transformed fold-change data are presented in Additional file 1.

### Real-time relative quantification polymerase chain reaction

For validation of microarray data, RNA samples from the 30-hour time point were converted to cDNA by means of the PowerScript (Clontech) reverse transcriptase in accordance with the recommended protocol with 1 μg of total RNA. Quantification of transcript levels was performed using SYBR Green chemistry on the ABI PRISM^® ^7900 HT Sequence Detection System (Applied Biosystems, Foster City, CA, USA). Polymerase chain reaction (PCR) amplification was carried out in 20 μl of reaction mixture consisting of 50 nM of primer pairs. The following primers were used for PCRs: cell division cycle 2 (CDC2), CCCAAATGGAAACCAGGAAG (forward), CGTTTGGCTGGATCATAGAT (reverse); cell division cycle 6 (CDC6), GGAAACCCGTTTGACAAAGG (forward), CGGGTGGGGTGTAAGAGAAGAATT (reverse); cell division cycle 28 protein kinase regulatory subunit 2 (CKS2), CTGATGTCTGAAGAGGAGTG (forward), GGAAGAGGTCGTCTAAAGAG (reverse); DNA replication helicase 2-like (DNA2L), GGATGGAACTGTTGGTGAAC (forward), ATTTAGTGAGGGCACACACC (reverse); ERβ, TCCATGCGCCTGGCTAAC (forward), CAGATGTTCCATGCCCTTGTTA (reverse); and β actin, ACCCACACTGTGCCCATCTACGAG (forward), TCTCCTTAATGTCACGCACGATTTCC (reverse). 18S rRNA Pre-developed TaqMan^® ^Assays by Applied Biosystems were used as reference gene.

### Patients and tumor specimens

The original patient material consisted of freshly frozen breast tumors that came from a population-based cohort of 315 women and that represented 65% of all breast tumors resected in Uppsala County, Sweden, from 1 January 1987 to 31 December 1989 [37,38]. The follow-up RNA expression profiling study was approved by the ethical committee at the Karolinska Institute, Stockholm, Sweden. Affymetrix (Santa Clara, CA, USA) oligonucleotide microarrays (U133A&B) were used to determine gene expression profiles in breast tumor samples. After exclusions based on RNA integrity and array quality control, expression profiles of 260 tumors were deemed suitable for further analysis [37]. To assess the role of ERβ-regulated cell cycle and DNA replication genes in breast tumor biology, we selected the 69 ER-positive tumors from patients who underwent adjuvant tamoxifen therapy (see Additional file 2). Clinico-pathological characteristics were derived from the patient records and from routine clinical measurements at the time of diagnosis as described elsewhere [37]. Histopathological re-examination and grading according to Elston-Ellis were performed by an experienced breast cancer pathologist without prior knowledge of selected therapies and outcomes. Gene expression data are presented as mean-centered log2-transformed normalized intensity for the probe set. The distances between expression profiles were calculated based on Pearson correlation, and the average-linkage method from the Eisen Cluster program was used to cluster genes and tumor samples. All cluster data were generated in the same way and using the same parameters to maintain the consistency of the analysis.

## Results

### Screen for ERβ-regulated E2-responsive genes

**Figure 1:**
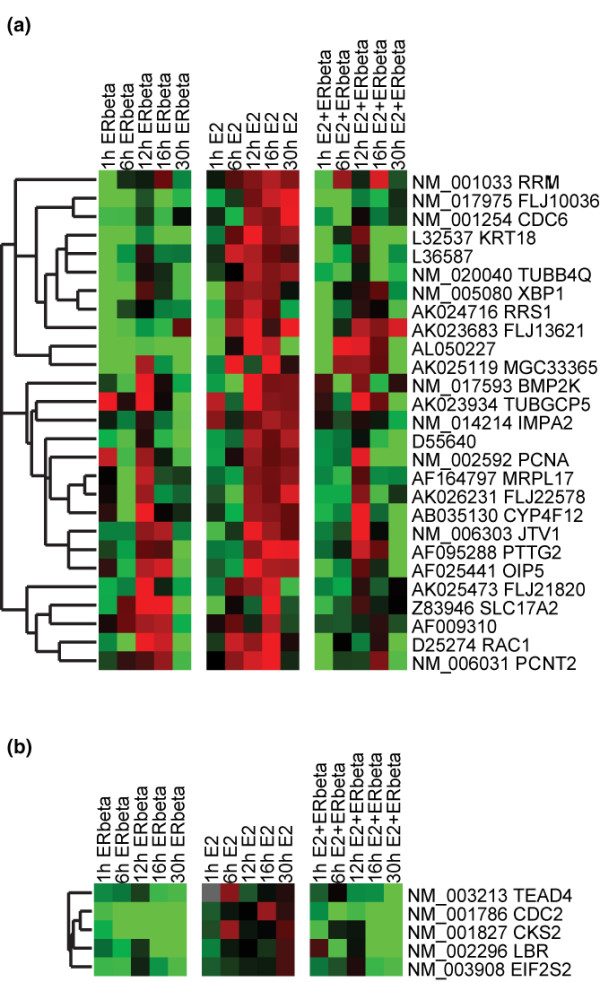
Two clusters of genes showing disruption of estrogen-responsive expression profiles by estrogen receptor beta (ERβ) overexpression. **(a) **The columns represent time points arranged in chronological order, and each row represents the expression profile of a particular gene. By convention, upregulated genes are indicated by red signals and downregulated genes are indicated by green. The magnitude of change is proportional to the brightness of the signal. (b) Second cluster of genes disrupted by ERβ overexpression.

ERβ expression was induced in the T-47Dbeta subline by TET withdrawal (see Materials and methods), and global gene expression profiles in the presence or absence of hormone were determined by human oligonucleotide microarrays (approximately 19,000 genes; Compugen USA, Inc.) at 1, 6, 12, 16, and 30 hours following induction. The preliminary expression data were subjected to a filter for genes that exhibited at least a 1.15-fold differential expression ratio between induced and treated samples and mock-treated (-E2)-uninduced (+TET) controls at each time point and in the same direction for three consecutive time points or in four out of the five time points. Eight hundred ninety-three upregulated genes and 728 downregulated genes met the selection criteria. These parameters were selected based on previously published time-course data and the ability to capture known responsive genes [28]. We then examined the expression profiles of responsive genes by hierarchical clustering. Two clusters of genes emerged which may explain the antagonistic activity of ERβ on cell growth. The first cluster contains genes whose expression levels were upregulated by E2 treatment but downregulated following ERβ expression alone or E2 treatment and ERβ expression (Figure 1a). Among the genes found within this cluster, *PCNA *(proliferating cell nuclear antigen) and *CDC6 *are both known to be involved in DNA replication and have been shown to be estrogen-responsive genes [29,39]. The second cluster includes *CDC2 *and *CKS2*, two cell cycle regulatory genes that were also downregulated by ERβ expression in the presence and absence of E2 (Figure 1b). The highly specific contrary regulation of a small number of genes, specifically the downregulation of genes involved in cell cycle regulation and DNA replication, presents a plausible mechanism by which ERβ or α/β heterodimers can block E2-induced cell proliferation.

### ERβ disrupts expression of cell cycle and DNA replication genes

To determine whether specific molecular pathways and processes are affected by ERβ expression, we assessed in an unbiased fashion whether the genes that were perturbed could be assigned to functional categories by gene ontology and whether genes of a specific function in cell proliferation were statistically significantly enriched, as measured by χ^2 ^tests, when compared to the frequency of these genes represented on the microarray (gene ontology annotated by Compugen USA, Inc.; see Additional File 3) (Table 1).

We first observed that ERβ in the presence of E2 did not significantly upregulate any specific functional class of genes. However, in the downregulated genes, we observed 1.7-fold (2.6% versus 1.5%) enrichment in the frequency of cell cycle genes (χ^2 ^*p *= 0.0241) and a 2.8-fold (1.4% versus 0.5%) enrichment in DNA replication genes (χ^2 ^*p *= 0.00175). We then further divided the 728 downregulated genes into expression profiles relative to E2 treatment alone into three models of interactions between ERβ and ERα. In the synergistic model, ERβ overexpression enhances E2 response, whereas in the attenuation model, ERβ diminishes the observed E2-regulated gene expression. When genes exhibit opposite E2-responsive regulation following ERβ expression, they are categorized under the antagonistic model of interaction between ERβ and ERα. All genes were assigned to these categories based on a cutoff of greater than (synergistic) or less than (attenuation) the E2 response or if the fold-change had an opposite (+ or -) notation (antagonistic) after log2 transformation. Table 2 shows the enrichment of responsive genes in the four cell proliferation/growth-associated functional categories. We found that cell cycle genes were over-represented (5.6-fold; χ^2 ^*p *= 0.00077) in the group described by the antagonistic model and that DNA replication genes were enriched 3.2-fold (χ^2 ^*p *= 0.0008) in the group that showed attenuation by ERβ of the E2-mediated transcriptional induction. Thus, it appears that ERβ can specifically regulate the proliferative effects of E2 and ERα by reducing the expression levels of components of the cell cycle and DNA replication machinery.

### Gene expression profiles in patient samples

We sought to address whether this *in vitro *effect of ERβ on proliferation could explain some of the heterogeneity in biological behavior of ERα-positive breast cancer. We posited that higher ERβ levels would be correlated with lower expression of these specific DNA replication and cell cycle genes and, in turn, would be associated with better clinical outcome. To test this hypothesis, we examined ERβ transcript levels in primary breast tumors with the expression profiles of ERβ-regulated cell cycle genes identified in our *in vitro *analysis and their association with clinical outcome.

**Figure 2:**
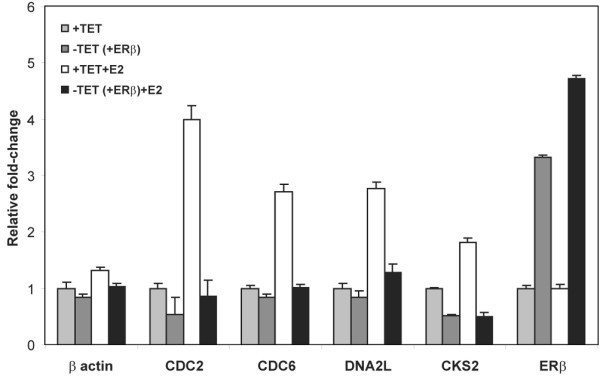
Validation of estrogen-responsive regulation of cell cycle and DNA replication genes suppressed by estrogen receptor beta (ERβ) overexpression by real-time quantitative polymerase chain reaction. *CDC2*, *CDC6*, *DNA2L*, and *CKS2 *were selected for further validation based on their significant correlation with ERβ transcript levels, and β actin expression was assessed as a negative control. Transcript levels in T-47Dbeta cells were measured at 30 hours following estrogen treatment (+E2) or mock treatment and under induction (-TET [+ERβ]) and non-induction (+TET) conditions. Relative fold-changes were calculated using the non-induced (+TET) samples as the reference. CDC2, cell division cycle 2; CDC6, cell division cycle 6; CKS2, cell division cycle 28 protein kinase regulatory subunit 2; DNA2L, DNA replication helicase 2-like; TET, tetracycline.

To study these *in vitro *observations in a biologically and clinically relevant context in which ERα is present and the function of estrogen-responsive pathways was targeted by anti-estrogen treatment, we specifically examined microarray expression data from 69 ERα-positive breast tumors from patients belonging to a previously described cohort [37,38] from Uppsala, who had received adjuvant tamoxifen therapy following surgery (see Materials and methods). We extracted the expression data of the six cell cycle and eight DNA replication genes (Table 2) that were downregulated by ERβ in the presence of E2 and classified the tumors based on their expression profiles. Two of the eight DNA replication genes were not found on the arrays used for the tumor studies, so only 12 genes in total were available for analysis. We first assessed whether ERβ expression was associated with a decrease in the expression of the ERβ-regulated cell cycle genes. When we examined these genes, we found that for the majority of them (10/12; 83%), there is negative correlation between ERβ transcript levels and the ERβ-responsive genes (Table 3). This is consistent with our *in vitro *findings that overexpression of ERβ leads to the downregulation of these genes. Four genes, *CDC2*, *DNA2L*, *CDC6*, and *CKS2*, were significantly (Student *t *test *p *< 0.05) associated with ERβ transcript levels in the microarray data. The estrogen-dependent upregulation of these genes and the suppression of their expression by ERβ overexpression in the cell line were further validated by semi-quantitative PCR (Figure 2) in cells treated for 30 hours with E2 to replicate prolonged estrogen exposure of breast tumor cells *in vivo*.

**Figure 3:**
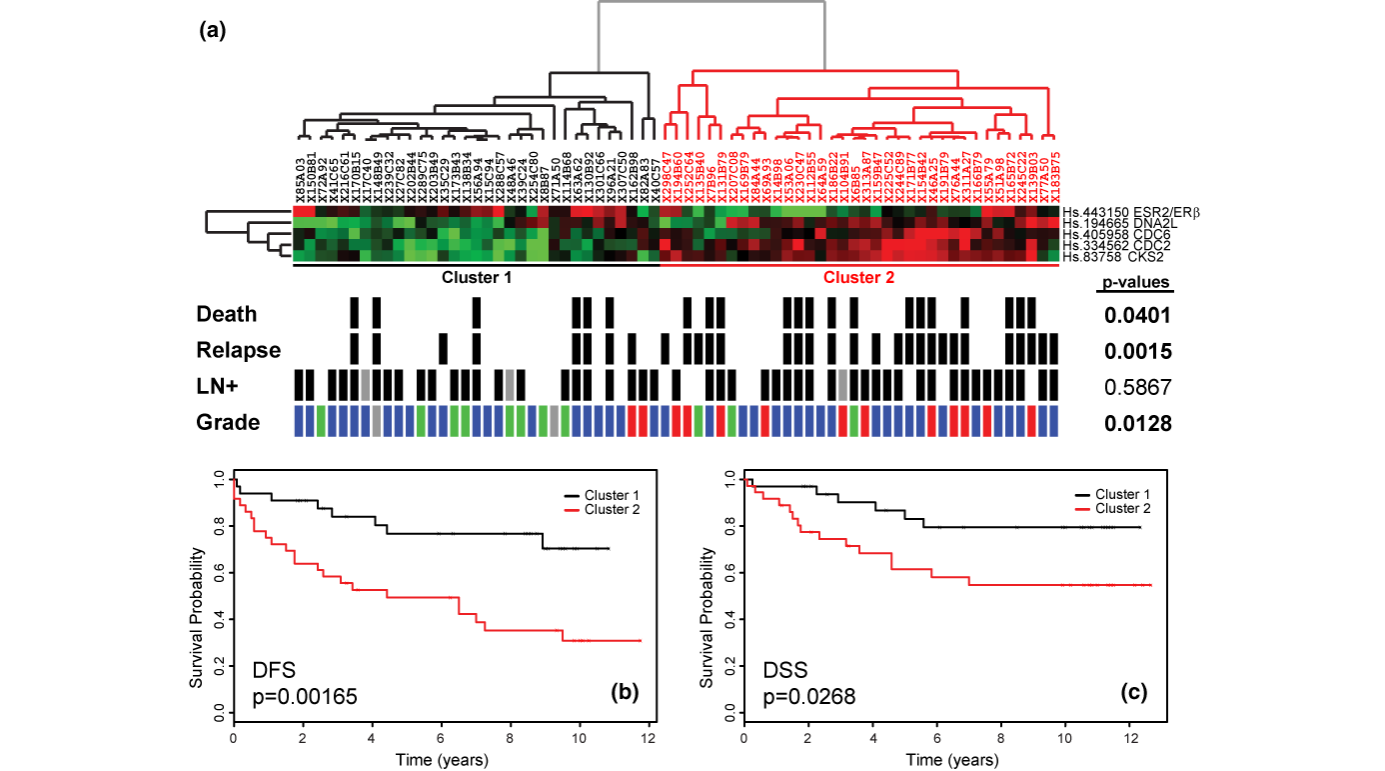
Clustering of 69 tumor samples by ERβ/ESR2, CDC2, CDC6, DNA2L, and CKS2 expression profiles is associated with clinical parameters and disease outcome. **(a) **Hierarchical clustering of tumor samples into two groups of high (black dendrogram; cluster 1) and low (red dendrogram; cluster 2) expression clusters. Clinical parameters, including relapse and death from breast cancer within 10 years of surgery and lymph node-positive (LN+) status, are indicated with a solid bar beneath each tumor sample. Tumor grade is indicated by colored bars; green, blue, and red bars denote grades 1, 2, and 3, respectively. Gray bars denote missing data. Significant distribution of clinical parameters between the two clusters was determined by Fisher exact tests, and *p *values were derived from the calculated distributions. **(b) **Kaplan-Meier plot of 10-year censored disease-free survival (DFS) curves for cluster 1 (black) and cluster 2 (red) patients is shown with the associated *p *values from likelihood-ratio analysis. **(c) **Kaplan-Meier plot of 10-year censored disease-specific survival (DSS) curves for cluster 1 (black) and cluster 2 (red) patients is shown with the associated *p *values from likelihood-ratio analysis. CDC2, cell division cycle 2; CDC6, cell division cycle 6; CKS2, cell division cycle 28 protein kinase regulatory subunit 2; DNA2L, DNA replication helicase 2-like; ERβ/ESR2, estrogen receptor beta.

We then focused our analysis on these four validated ERβ-regulated genes (*CDC2*, *CKS2*, *CDC6*, and *DNA2L*) with significant correlation with ERβ expression in the tumors as the functional ERβ-responsive gene set for subsequent clustering and associations with clinical parameters (Figure 3). With the expression of the ERβ-responsive gene set, tumor samples were clustered (average linkage) into two major groups with statistically significant association with 10-year relapse (Fisher exact *p *= 0.0015), death (Fisher exact *p *= 0.0401), and tumor grade (Fisher exact *p *= 0.0128) as compared to the distribution of the parameters tested in the study population.

**Figure 4:**
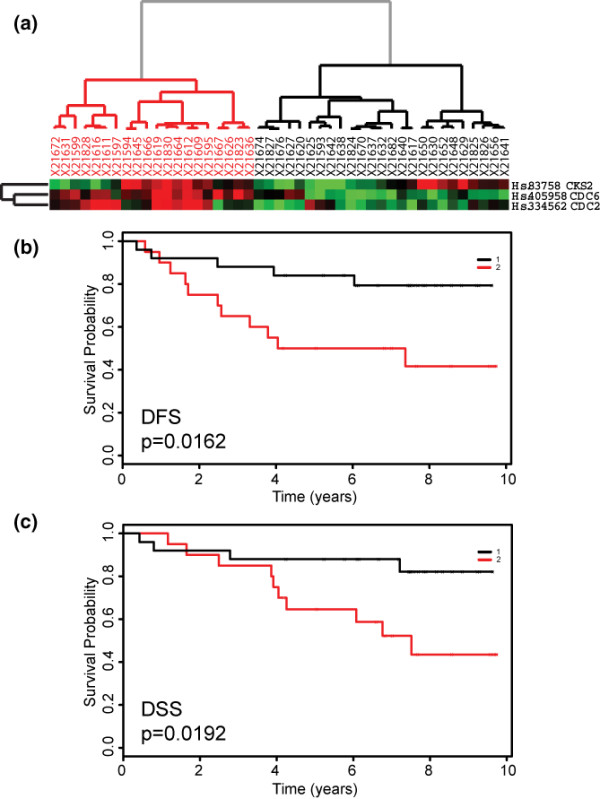
Clustering of 45 previously published ERα-positive tumor samples [40] confirms that ERβ-responsive gene set expression profile is associated with disease outcome. **(a) **Hierarchical clustering of tumor samples into two groups of high (black dendrogram) and low (red dendrogram) ERβ expression clusters. **(b) **Kaplan-Meier plot of disease-free survival (DFS) curves for high (black) and low (red) expression patients is shown with the associated *p *values. **(c) **Kaplan-Meier plot of disease-specific survival (DSS) curves for high (black) and low (red) expression patients is shown with the associated *p *values. Due to the absence of the corresponding probes on the arrays used by Sotiriou and colleagues [40], ERβ and DNA2L transcript levels were not assessed. DNA2L, DNA replication helicase 2-like; ER, estrogen receptor.

Using disease-free survival (DFS) (Figure 3b) and disease-specific survival (DSS) (Figure 3c) as clinical endpoints, we performed Kaplan-Meier survival analysis on the two patient clusters to ascertain the correlation between the molecular signature and disease outcome. Patients with relatively higher levels of ERβ and lower expression of the gene set transcript (Figure 3a, cluster 1; Figure 3b,c, black curves) have significantly improved outcomes compared to the group with lower levels of ERβ and higher responsive gene set transcript levels (Figure 3a, cluster 2; Figure 3b,c, red curves) for both endpoints, DFS (likelihood-ratio *p *= 0.0017) and DSS (likelihood-ratio *p *= 0.0268). As expected, we did not observe any significant association of the ERβ-responsive gene set expression profiles with disease outcome in 37 ERα-negative tumors from the same patient population (data not shown). These results were further validated in an independent dataset of 45 ERα-positive tumors from patients who also underwent endocrine therapy [40]. In this independent dataset of British patients, the ERβ-responsive gene set expression profiles also identified patients in high and low ERβ gene set expression groups (Figure 4a) and to poor and good outcome groups for DFS (Figure 4b, likelihood-ratio *p *= 0.0162) and DSS (Figure 4c, likelihood-ratio *p *= 0.0192), respectively. Our findings indicate that the observed *in vitro *activity of ERβ on downstream target genes is directly recapitulated in primary breast tumors and has utility in further demarcating prognostic and therapeutic subgroups in the clinical setting. In addition, these results implicate the selective ERβ transcriptional effects as identified *in vitro *in defining clinical behavior of human breast tumors.

## Discussion

Original characterization of ERβ function indicated its ability to form homodimers and heterodimers (with ERα) and to bind the same consensus ERE sequences in electrophoretic mobility shift experiments [9]. However, the biological effects of ERβ are considerably different from those of ERα: ERβ expression alone, unlike hormone treatment, was not sufficient to drive cell proliferation [28]. In cell lines, overexpression of ERβ inhibited E2-driven and ERα-mediated cell proliferation [28]. In our microarray screen for transcriptional effects of ERβ expression in ERα-positive cells, statistical analysis indicated a significant enrichment of genes from two cell growth-associated functional categories within the population of genes downregulated by ERβ in the presence of E2 (Tables 2 and 3). However, because both receptor subtypes are present in the cells, it is not clear from the experimental system whether the observed transcriptional effects were due to the activity of ERβ homodimers or α/β heterodimers.

One of the difficulties in assessing the biological significance of such associations between *in vitro *gene expression changes and the cellular response is that a number of genes coordinately expressed (for example, the ERβ-responsive gene set) may be involved. One-by-one targeting of all responsive genes not only is experimentally problematic but also raises the question of relevance given that an entire gene set may need to be altered. We pursued an alternative approach to determining the *in vivo *relevance of these findings in the engineered cell line by exploring the association of ERβ transcript levels and the key ERβ-suppressed cell cycle and DNA replication genes in disease outcome of patients with breast cancer. We hypothesized that if our *in vitro *findings were biologically important, their configuration should be captured in clinical tumors and their biological impact should recapitulate that found in cell culture.

To this end, we first found significant association of the expression profiles of four genes, *CDC2*, *CKS2*, *DNA2L*, and *CDC6*, with ERβ transcript levels in the breast tumors, consistent with the inverse correlation (high ERβ; low cell cycle/DNA replication genes) observed in the cell line model. *CDC2 *is a cyclin-dependent kinase that regulates G_1_-S and G_2_-M phases of the cell cycle; *CKS2 *encodes the essential regulatory subunit of cyclin-dependent kinases; and *CDC6 *and *DNA2L *function in the pre-replication complex formation and unwinding of replicating DNA, respectively. Tumor samples were then hierarchically clustered into two distinct groups, using the expression profiles of this four-gene set and ERβ (Figure 3). We found significant association of the patient groups with tumor grade (which we consider a clinical surrogate for cell cycle and DNA replication) and with 10-year relapse and death. Moreover, the patient cluster with high ERβ and low ERβ downstream gene expression has significantly higher probability of DFS and DSS over time as compared to those with the opposite profiles. These results suggest that the ERβ-regulated genes identified in our *in vitro *studies are also involved in the biology of primary malignant breast tumors and reveal protective effects of ERβ in disease prognosis. That this effect is limited to the ERα-positive subgroup is also consistent with our observations that the main ERβ effect is in the presence of ERα and the E2 ligand. Previously, we reported that ER-positive tumors exhibited significant differences detected by expression profiling associated with variances in overall survival [40]. Using the same dataset, we assessed the impact of ERβ and its unique downstream responsive gene set and found that this minimal gene set alone could account for the majority of the survival differences [40]. This suggests that ERβ status may be a major driver for clinical heterogeneity in ER-positive tumors.

Our findings are consistent with a recent report by Hopp and colleagues [41] in which they found that low ERβ protein levels are associated with poor DFS and overall survival in ER-positive patients treated with adjuvant tamoxifen therapy. In contrast, another group reported the lack of association between ERβ transcript levels and disease outcome following endocrine therapy in a prospective study [42]. One possible explanation for the discrepancy between the prospective study and the previous protein level study and the microarray expression data reported here is that protein levels and expression profiles of downstream ERβ-regulated genes are a more proximal measure of ERβ activity than the transcript levels alone.

A reasonable question is whether the ERβ downstream genes harbor the transcriptional control elements associated with the ERs (that is, EREs). Our assessment of the 5' regulatory regions of the four key downstream genes *CDC2*, *CKS2*, *DNA2L*, and *CDC6 *showed no evidence for an acceptable ERE (data not shown). This raises the possibility either that the expression of these genes involves trans-elements such as other transcription factors induced by ERβ or that ERβ is acting as a co-factor for other transcription factors such as AP-1 [43,44]. In an earlier microarray study of estrogen-responsive genes, Lobenhofer and colleagues [39] observed an increase in the expression of a number of DNA replication fork genes, including those that have been shown to be regulated by the E2F transcription factor. Indeed, a number of studies showed that E2F is involved in regulating the expression of *CDC6 *[45-47]. These findings suggest that ERβ may affect cell proliferation, in part, through interactions with the E2F transcriptional regulatory pathways.

## Conclusion

Taken together, our observations point to specific inhibitory effects of ERβ on hormone-dependent breast cancer cell proliferation and also suggest that molecules directly targeting ERα/ERβ interactions or ERβ mimics could be a novel strategy to develop estrogen response modifiers for the management of ERα-positive breast tumors.

## Abbreviations

CDC2 = cell division cycle 2; CDC6 = cell division cycle 6; CKS2 = cell division cycle 28 protein kinase regulatory subunit 2; DNA2L = DNA replication helicase 2-like; Cy = cyanine; DBD = DNA-binding domain; DCCFBS = dextran-coated charcoal-treated fetal bovine serum; DFS = disease-free survival; DMEM = Dulbecco's modified Eagle's medium; DSS = disease-specific survival; ER = estrogen receptor; ERE = estrogen response element; FBS = fetal bovine serum; PCR = polymerase chain reaction; TET = tetracycline.

## Competing interests

J-ÅG is cofounder, deputy board member, consultant, and shareholder of Karo Bio AB (Huddinge, Sweden). A patent application covering the discoveries reported in this article has been filed by C-YL, ETL, and the Genome Institute of Singapore (Republic of Singapore). The other authors declare that they have no competing interests.

## Authors' contributions

C-YL conceived the study, drafted the manuscript, and performed the cell line microarray experiments. AS conceived the study, generated the cell line, and performed treatments and RNA extraction. J-ÅG and ETL conceived the study and drafted the manuscript. SK generated the cell line and performed treatments and RNA extraction. SLK, JBST, and JST planned and performed the validation experiments. VBV assisted with data analysis. LDM assisted with data analysis and performed the microarray study of patient samples. JB collected the patient samples from an Uppsala, Sweden cohort and performed the microarray study of patient samples. JS performed the microarray study of patient samples. All authors read and approved the final manuscript.

## Supplementary Material

Additional file 1 Cell line estrogen receptor beta overexpression and E2 treatment microarray data with log2-transformed fold-change data as compared to the untreated uninduced control at each time point. Click here for file

Additional file 2 Uppsala patient information, including estrogen receptor (ER) beta transcript levels and ER alpha protein levels. Click here for file

Additional file 3 Gene ontology annotations (2002 version) made by Compugen USA, Inc. (Jamesburg, NJ, USA) for probes represented on the microarrays used for the cell line study. Click here for file

## Figures and Tables

**Table 1:** Statistics of key gene ontology categories

Gene function	Percentage total	χ^2 ^*p *value
E2 + ERβ upregulated		
Apoptosis	1.1% (10/893)	0.18590
Cell cycle	1.5% (13/893)	0.82478
Cell proliferation	0.4% (4/893)	0.41984
DNA replication	0.4% (4/893)	0.80576
E2 + ERβ downregulated		
Apoptosis	0.4% (3/728)	0.31923
Cell cycle	2.6% (19/728)	0.02471
Cell proliferation	0.3% (2/728)	0.91690
DNA replication	1.4% (10/728)	0.00175
All genes on array		
Apoptosis	0.7% (138/18,912)	-
Cell cycle	1.5% (293/18,912)	-
Cell proliferation	0.3% (56/18,912)	-
DNA replication	0.5% (96/18,912)	-

ERβ, estrogen receptor beta.

**Table 2:** Statistics of key gene ontology categories by mechanism of regulation in 728 E2 + ERβ downregulated genes

Gene function	Percentage total	χ^2 ^*p *value
'Synergistic'		
Apoptosis	0% (0/129)	0.33018
Cell cycle	0.8% (1/129)	0.47730
Cell proliferation	0% (0/129)	0.53595
DNA replication	0.8% (1/129)	0.67051
'Attenuation'		
Apoptosis	0.4% (2/492)	0.40303
Cell cycle	1.8% (9/492)	0.11725
Cell proliferation	0.2% (1/492)	0.70713
DNA replication	1.6% (8/492)	0.00080
'Antagonistic'		
Apoptosis	0.9% (1/107)	0.80404
Cell cycle	8.4% (6/107)	0.00077
Cell proliferation	0.9% (1/107)	0.22828
DNA replication	0.9% (1/107)	0.53640
All genes on array		
Apoptosis	0.7% (138/18,912)	-
Cell cycle	1.5% (293/18,912)	-
Cell proliferation	0.3% (56/18,912)	-
DNA replication	0.5% (96/18,912)	-

ERβ, estrogen receptor beta.

**Table 3:** Association with estrogen receptor beta expression in 69 breast tumors

UniGene ID^a^	Symbol	Correlation	*p *value
Hs.334562	*CDC2*	-0.30798	0.00151
Hs.194665	*DNA2L*	-0.22919	0.01522
Hs.405958	*CDC6*	-0.16138	0.02774
Hs.83758	*CKS2*	-0.21564	0.04152
Hs.156346	*TOP2A*	-0.11536	0.07061
Hs.436806	*RAMP*	-0.20123	0.07442
Hs.436102	*FLJ12484*	0.05731	0.08202
Hs.115474	*RFC3*	-0.19135	0.11683
Hs.139226	*RFC2*	-0.09613	0.12292
Hs.135665	*GAS2*	0.05865	0.24376
Hs.142179	*INCENP*	-0.03967	0.29616
Hs.234896	*GMNN*	-0.07458	0.36950
Hs.22116	*CDC14B*^b^	N/A	N/A
N/A	*PRO0245*^b^	N/A	N/A

^a^UniGene Build 175; ^b^not found on the arrays. N/A, not applicable.
